# Supplementary material for: Colombian Scorpion Centruroides margaritatus: Purification and Characterization of a Gamma Potassium Toxin with Full-Block Activity on the hERG1 Channel
Source: Toxins (Basel). 2021 Jun 8;13(6):407. doi: 10.3390/toxins13060407 (PMC8273696; doi:10.3390/toxins13060407)
Supplement: Supplementary file 1 [file toxins-13-00407-s001.zip › toxins-1239397 - supplementary corrected.pdf]

Supplementary Materials: Colombian Scorpion *Centruroides margaritatus*: Purification and Characterization of a Gamma Potassium Toxin with Full-Block Activity on the hERG1 Channel

José Beltrán-Vidal, Edson Carcamo-Noriega, Nina Pastor, Fernando Zamudio-Zuñiga, Jimmy Alexander Guerrero-Vargas, Santiago Castaño, Lourival Domingos Possani and Rita Restano-Cassulini

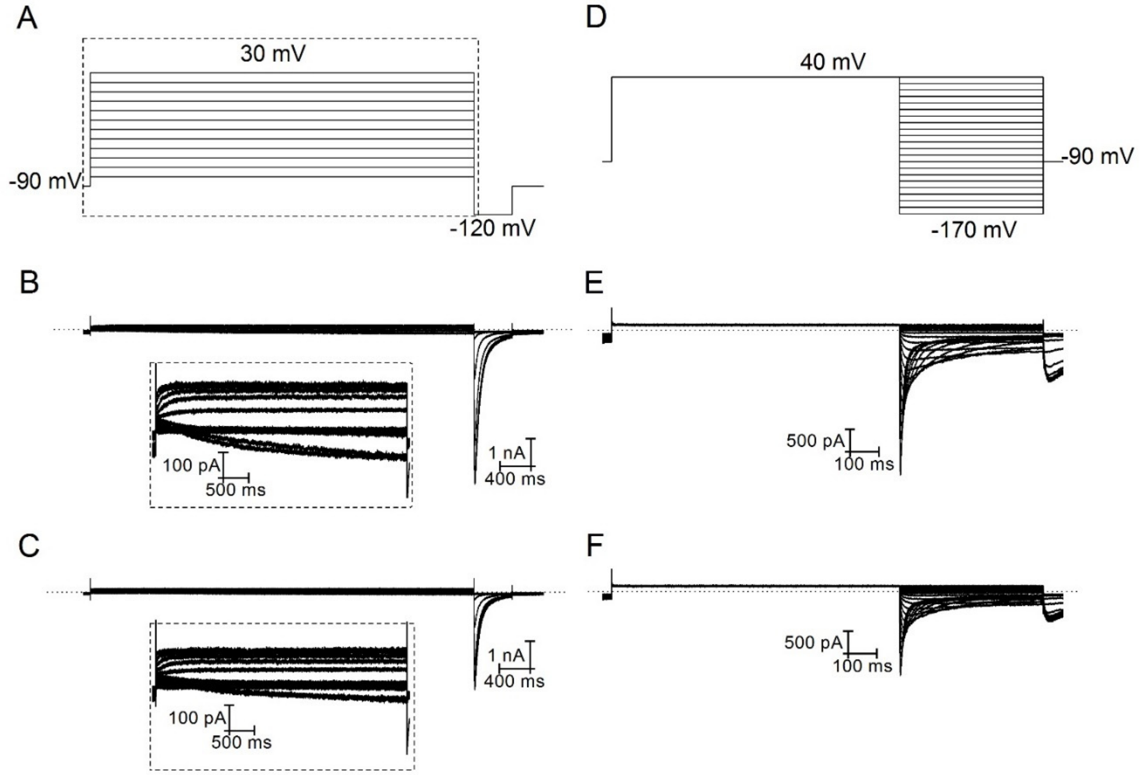

Figure S1. Whole hERG1 currents recorded during the activation and inactivation protocols depicted in **A** and **D** respectively, before (**B** and **E**) and after 1–2 min of 4 nM CmERG1 exposition (**C** and **F**). During the depolarization steps, the channels activate, but more quickly they inactivate resulting in a current considerably smaller if compared with the tail current recorded during the subsequent hyperpolarizing step. In the dash panels (**B** and **C**) the outwards currents are showed amplified. Currents in **B**–**F** are representative of $n = 3$.
